# Congruence of Additive and Non-Additive Effects on Gene Expression Estimated from Pedigree and SNP Data

**DOI:** 10.1371/journal.pgen.1003502

**Published:** 2013-05-16

**Authors:** Joseph E. Powell, Anjali K. Henders, Allan F. McRae, Jinhee Kim, Gibran Hemani, Nicholas G. Martin, Emmanouil T. Dermitzakis, Greg Gibson, Grant W. Montgomery, Peter M. Visscher

**Affiliations:** 1 University of Queensland Diamantina Institute, University of Queensland, Princess Alexandra Hospital, Brisbane, Queensland, Australia; 2 The Queensland Brain Institute, University of Queensland, Brisbane, Queensland, Australia; 3 Queensland Institute of Medical Research, Brisbane, Queensland, Australia; 4 School of Biology and Centre for Integrative Genomics, Georgia Institute of Technology, Atlanta, Georgia United States of America; 5 Department of Genetic Medicine and Development, University of Geneva Medical School, Geneva, Switzerland; King′s College London, United Kingdom

## Abstract

There is increasing evidence that heritable variation in gene expression underlies genetic variation in susceptibility to disease. Therefore, a comprehensive understanding of the similarity between relatives for transcript variation is warranted—in particular, dissection of phenotypic variation into additive and non-additive genetic factors and shared environmental effects. We conducted a gene expression study in blood samples of 862 individuals from 312 nuclear families containing MZ or DZ twin pairs using both pedigree and genotype information. From a pedigree analysis we show that the vast majority of genetic variation across 17,994 probes is additive, although non-additive genetic variation is identified for 960 transcripts. For 180 of the 960 transcripts with non-additive genetic variation, we identify expression quantitative trait loci (eQTL) with dominance effects in a sample of 339 unrelated individuals and replicate 31% of these associations in an independent sample of 139 unrelated individuals. Over-dominance was detected and replicated for a *trans* association between rs12313805 and ETV6, located 4MB apart on chromosome 12. Surprisingly, only 17 probes exhibit significant levels of common environmental effects, suggesting that environmental and lifestyle factors common to a family do not affect expression variation for most transcripts, at least those measured in blood. Consistent with the genetic architecture of common diseases, gene expression is predominantly additive, but a minority of transcripts display non-additive effects.

## Author Summary

Gene expression levels are known to influence common disease susceptibility in humans, with GWAS significant SNPs frequently found in regulatory regions. The expression levels of most genes are influenced by genetic variants, often located close to the gene itself. Expression Quantitative Trait Loci (eQTL) mapping studies have been very successful in identifying SNPs associated with expression levels; however, little is currently known about the extent of additive and non-additive genetic variance and the role of common environment on gene expression. Here we report a comprehensive study of the sources of genetic and non-genetic variation for gene expression levels using both pedigree and genotype information. We show that the majority of transcripts exhibit only additive genetic variance with congruence from independent methods using pedigree and genotype approaches. However, there are a small number of probes whose expression levels are influenced by non-additive genetic variance. For some of these probes we identify SNPs acting in a dominant and over-dominant manner that replicate in an independent sample. Surprisingly, only 17 probes exhibit significant levels of common environmental effects, suggesting that environmental and lifestyle factors common to a family do not affect expression variation for most transcripts, at least those measured in blood.

## Introduction

Understanding the nature of genetic variation for complex traits, including disease, is important in human medicine, evolutionary biology and plant and animal breeding. Both the nature of complex trait variation, including the importance of non-additive genetic variation, and its dissection into contributions from individual genetic loci, have been debated for a century [1]–[5]. Traditionally, inference about genetic variation for complex traits comes from the resemblance (or recurrence risk) of relatives but more recently genotyping and sequencing technologies have been developed that allow the attribution of genetic variation to specific loci. Recently, the debate on genetic variation has focussed on ‘missing heritability’ for human disease, the discrepancy between estimates of heritability from pedigree data and the cumulative variation explained by validated associated DNA variants. Many explanations of missing heritability have been proposed in the literature, including that pedigree estimates of narrow sense heritability may be inflated due to epistatic variance [5], causal variants not being in sufficient linkage disequilibrium with common SNPs because they are rare [6] and effect sizes too small to be detected with genome-wide significance [7].

Gene expression is an important complex trait because of increasing evidence of its correlation with disease susceptibility [8]–[11]. It is also an ideal model trait for genetic dissection: it can be measured genome-wide using array or sequencing methods so for each sample thousands of expression phenotypes can be obtained and analysed. Almost all studies looking at genetic variation influencing transcript levels in humans have done so using an additive model, however, studies in several model organisms have identified a substantial fraction of genes with non-additive or dominance inheritance patterns [12]–[15]. If gene expression variation is to be utilized more fully to inform on the biological mechanisms leading to disease susceptibility [11], then knowledge of the inheritance patterns and resemblance between individuals is clearly important.

In this study, we combine the power of pedigree and SNP-based designs to quantify and dissect the contribution of additive and non-additive variation using gene expression on 17,994 probes measured on 862 individuals from 312 nuclear families (the Brisbane Systems Genetics Study, BSGS) [16]. We find strong evidence and consistency for prevailing additivity, but also detect and replicate dominance variation and dominant SNP effects for a number of probes. We detect and replicate a single over-dominant locus where the heterozygous genotype at a SNP is associated with increased expression at a gene that is 4MB downstream.

## Results

We investigated the quantitative genetic architecture of gene expression using two orthogonal approaches. The first involved the decomposition of phenotypic variance using a family based analysis. In the second, additive and non-additive SNP effects were estimated from unrelated individuals within BSGS. We followed up associations with replication in an independent sample (Centre for Health Discovery and Well Being [17], CHDWB) of 139 European Americans (CHDWB_EA). By design, the estimates of variance components from pedigree data are independent of those using SNP associations.

### Family-based analysis of genetic variation

**Figure 1:**
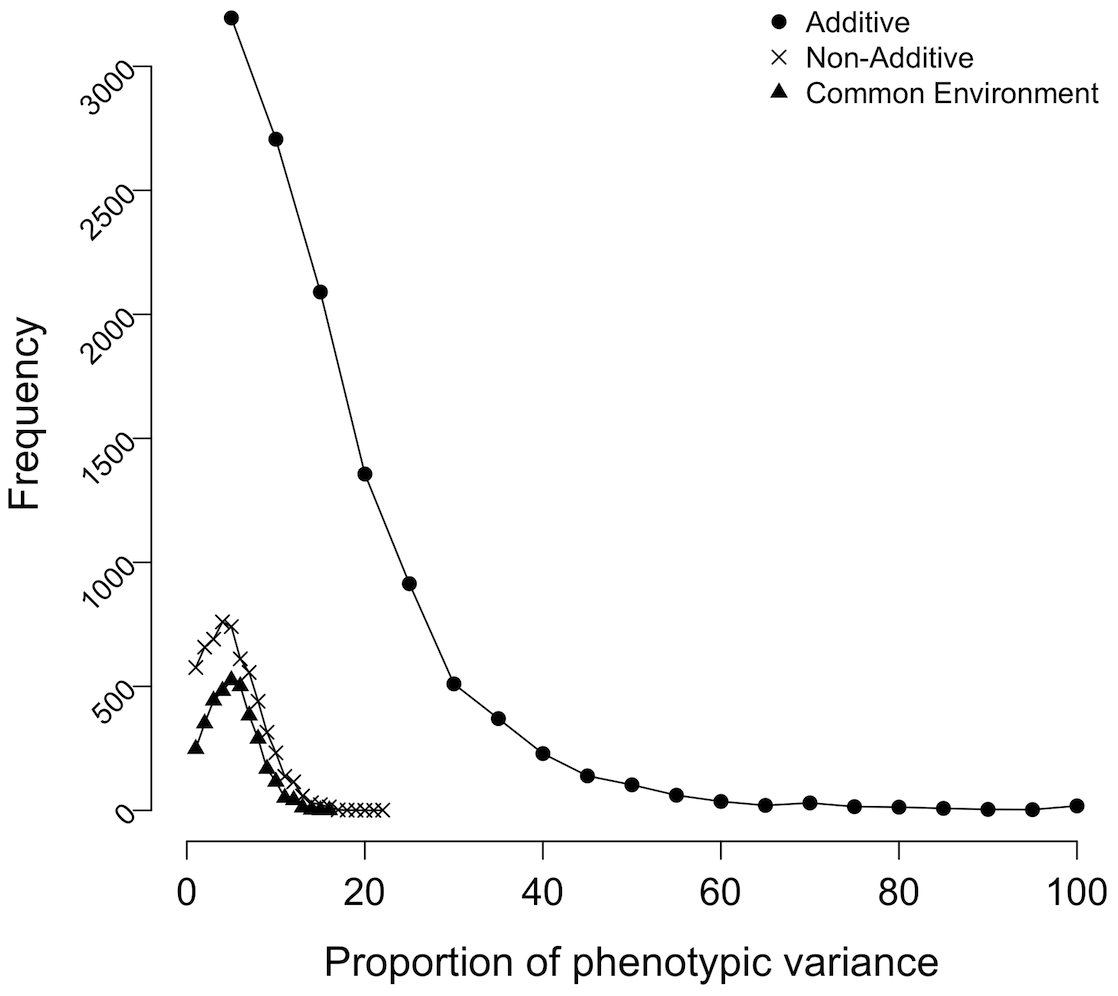
Components of variation. Distributions showing the proportion of phenotypic variance attributable to additive genetic (*h^2^*), non-additive genetic (*d^2^*) and common family (*f^2^*) effects. Only probes whose estimates are greater than zero are included. The distributions for all probes are given in Figure S1. Estimates of *h^2^* and *d^2^* were obtained by fitting an model [1], whist *f^2^* estimates were obtained from model [3].

Additive (*V_a_*) and non-additive (*V_d_*) genetic variance components were estimated for RNA expression levels measured at 17,994 probes on 862 individuals across 312 families in BSGS, using restricted maximum likelihood. Of 17,994 probes that passed QC (methods), 14,753 (82%) had narrow-sense heritability (*h^2^*) estimates >0 (Figure 1 and Figure S1). Under the assumption of no additive genetic variance for any probe, we would expect to observe ∼9,000 probes (∼50%) with estimates of *V_a_* = 0 as our estimates are constrained to non-negative. Here we find that the proportion of probes with *h^2^* estimates >0 is 0.82. Therefore, accounting for sampling variance of *h^2^* estimates we expect the proportion of probes that have true heritable variation is 2(0.82–0.50) = 0.64, in concordance with other studies [18], [19]. Although non-additive variance is more difficult to detect due to higher sampling variances and confounding with estimates of additive and shared environmental variance [2], [3], we find 5,798 probes (32%) have a non-zero estimate of *V_d_* (Figure 1 and Figure S1). The observation that greater than 50% of probes have a zero estimate of *V_d_* is due to the estimation of *V_d_* within a model that jointly estimates *V_a_* (Text S1 and Figure S2). The results for dominance are consistent with the majority of probes not displaying dominance variation. The conclusions from the number of non-zero estimates are also consistent with that from employing a false discovery rate (FDR = 0.05) approach: 11,957 (66%) of probes had an estimate of additive variation significantly different from zero, while for only 960 (5.3%) was dominance variation significantly different from zero (corresponding *P*-value thresholds, 1.3e^-5^ and 9.7e^-6^, respectively) (Table 1). A small number of probes (678, or 3.7%) had both significant additive and dominance variation. For those probes, the additive component was much larger than the dominance component (Figure S3). Hence, by jointly estimating additive and dominance variation in pedigrees on ∼18,000 genome-wide RNA transcripts, we conclude that the majority of probes display genetic variation, most of which appears additive (Figure S3).

**Table 1:** Estimates of additive and non-additive genetic components of transcript expression levels estimated using genetically orthogonal approaches in related and unrelated individuals.

Pedigree analysis		SNP analysis on unrelated individuals		
		Number of probes with a significant eQTL		
Variance components	N Significant (FDR 0.05)	Additive effect only	Additive + Dominance effects	Over-Dominance effect only
**V_A_** only	11,279	3,364 (1,017)	27 (4)	0
**V_A_** and **V_D_**	678	243 (68)	113 (19)	1 (0)
**V_D_** only	282	7 (1)	61 (8)	6 (1)

Significance of variance components were determined at a study-wise FDR = 0.05, corresponding top-value thresholds of 1.3e-5 and 9.7e-6 for additive and dominance variation, respectively. The numbers of eQTL that replicate in CDHWB_EA are given in brackets.

### Non-genetic familial variation

We next estimated the proportion of phenotypic variation attributable to familial non-genetic factors (*V_f_*). The proportion of phenotypic variance attributed to within families was partitioned by inclusion of a family term alongside the additive genetic relationship term (see equation [3] in methods). In total 3,373 probes had a non-zero estimate of 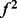 (Figure 1), which is consistent with estimates from expression levels measured in skin and fat tissue [18]. As with *V_d_*, the high proportion of zero estimates is likely due to the estimation of *V_f_* jointly with *V_a_*. However, non-zero estimates do not fully represent the true underlying level of common family variance as a proportion of non-zero estimates are expected by chance due to sampling error (Figure S4 and Text S2). This is important when we consider that the expected sampling variance of *V_d_* and *V_f_* are greater than *V_a_* [2], [3]. Indeed, at an FDR 0.05 (corresponding to *p*<2.3e^-7^) only 17 probes (in 17 genes) (Table S1) show a significant estimate of non-genetic familial variation. On average, the phenotypic correlations between parent pairs (n = 71) for probes with significant *V_f_* were 4 standard deviations above the mean correlation of all probes (Figure S5). We investigated shared biological functionality among the 17 genes by performing a GO term [20] enrichment analysis using GOEAST [21]. No GO terms were found to be significantly enriched suggesting that common environmental effects that influence the transcript levels act independently of a shared biological network. A possible and likely explanation is that there are numerous environmental effects that independently influence the transcription of specific genes [22], [23]. From these analyses we conclude that on average, environmental factors shared by all family members do not have a strong effect on gene expression measured in blood samples.

### Population-based analysis

We then performed a global eQTL analysis of additive and dominance effects, associating the 17,994 expression traits with SNP genotypes in a dataset of 339 unrelated individuals drawn from BSGS. In total, we identify a total of 5,033 eQTL [FDR 0.05, corresponding to *p*<4.8e^−4^ (*cis*) and *p*<6.2e^−10^ (*trans*)] with an additive effect across 3,364 probes (Table 1 and Table 2). The majority (84%) of these eQTL are located in *cis*-regions and on average the top eSNP (SNP with the strongest association) explained 11.3% of the phenotypic variance of the probe with which it is associated. Our analysis of dominance eQTL fits a model that includes a dominance effect (*d*) as well as the main additive effect. From this analysis we identify 208 eQTL (179 *cis*-acting and 29 *trans*-acting) that have a study-wide significant [FDR 0.05, corresponding to *p*<4.1e^−4^ (*cis*) and *p*<4.7e^-10^ (*trans*)] dominance effect, including 7 with over-dominance. Despite the power to detect dominance-acting eQTL being considerably lower than that to detect additive effects in SNP-trait association studies [24], we sought to replicate the dominance eQTL in an independent sample of 139 individuals. Of the 208 eQTL with dominance effects, 32 replicated at *p*<2.4e^−4^ = 0.05/208, the Bonferoni threshold (Table 1). For the remaining 176 SNPs not significant in the replication data we tested for differences in their estimates of *d* from CHDWB_EA, comparing the groups of SNPs that were +*d* against −*d* from BSGS (Figure S6). The significant difference between the +*d* and −*d* groups (*P* = 0.0031) indicates that these loci are enriched for dominance effects.

**Table 2:** eQTL identified from an additive (1df) test (FDR = 0.05).

		Conditional eQTL analysis		
	eQTL	Second	Third	Fourth
N probes	3,364	1,376	217	76
*Cis* (+/−1MB TSS)	84%	88%	84%	72%
*Trans*	16%	12%	21%	38%

FDR 0.05 level corresponds to *P*–value thresholds 4.8e-4 (*cis*) and 6.2e-10 (*trans*). Multiple eQTL were identified from a series of consecutive conditional analyses, up to the maximum of 4 independent eQTL.

**Figure 2:**
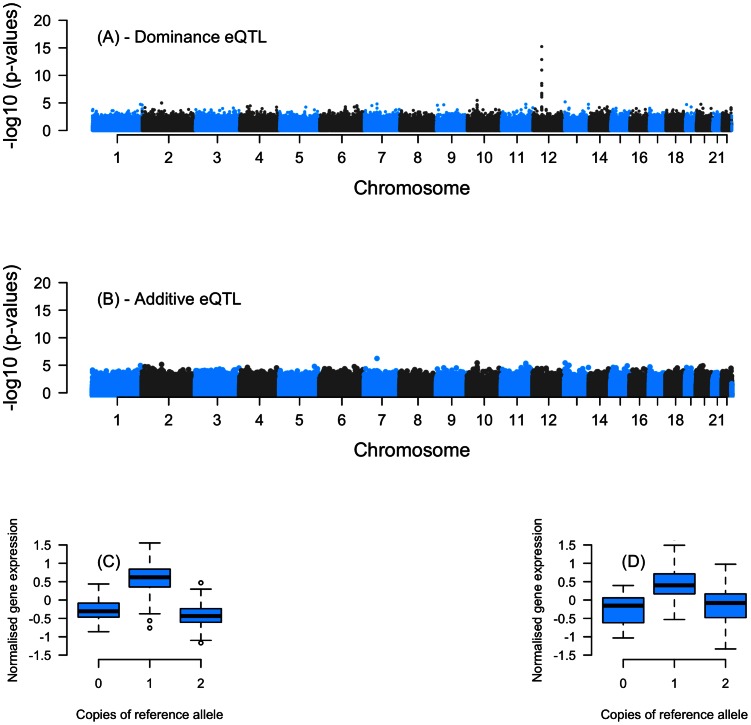
Association plots showing the −log10 *P*-values for SNPs tested against transcript expression levels of ILMN_1789596 probe in ETV6. (a) shows the *P*-values for the dominance component of a 2df additive and dominance model and (b) P-values from an additive only 1df model. A genome-wide significant dominance only association is located on chromosome 12 (p12.3). The genotype-phenotype map for the top eSNP (rs12313805) (*p* = 1.12e^−16^) is given in (c). The over-dominance association was replicated (p = 2.54e^−9^) in an independent dataset (CDHWB_EA). (d) is the genotype-phenotype maps for rs12331805 in CDHWB_EA. The MAF for rs12313805 in BSGS and CDHWB_EA were 0.32 and 0.45, respectively.

Of the six associations with significant over-dominance in BSGS, we replicate the association between rs12313805 (chr12:16,523,922; hg19) and a probe in ETV6 (TSS chr12:11,938,923; hg19), an ETS family transcription factor (Figure 2). Individuals carrying the heterozygous genotypes (A/G) for rs12313805 have an up-regulation of ILMN_1789596, the probe in ETV6, compared to individuals with the two homozygous genotypes that show no significant difference in expression levels. The average fold difference between heterozygous and homozygous individuals is 2.1 and 1.6 in BSGS and CHDWB_EA, respectively. One possible explanation for the observed over-dominance association is that it is caused by two tightly linked SNPs with additive effects in opposite allelic directions. To investigate this possibility we analysed a 10MB imputed (against 1000 Genomes V1.3) region, +/−5MB of rs12313805, for additive and non-additive associations (Figure S7). In this region there are no two SNPs with additive effects large enough that should they be in opposite directions, could combine to cause a spurious over-dominance association of the magnitude observe here. Furthermore, we performed a haplotype analysis using a three-SNP sliding window and looking at additive and non-additive haplotype associations [25]. Only haplotypes whose association models included non-additive terms showed significant associations (Figure S7).

To characterise the network effects of large *cis*-eSNP we calculated the inverse covariance matrix (**V^−1^**) [26], otherwise known as the precision matrix, of the gene expression levels for the 17,994 probes in the dataset of unrelated individuals. Because the normalised expression data follows a multivariate normal distribution, element [*i, j*] of **V^−1^** represents the partial correlation between probes *i* and *j*, conditional on remaining probes. The matrix is sparse with non-zero elements representing conditional correlations between probes [27]. For the 10 probes with eSNP that explain the largest proportion of 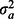 (Table 3) (hereon termed primary probes), we extracted a list of their conditionally correlated probes based on non-zero elements in V^−1^. For each of the conditionally correlated probes we extracted their association with the eSNP for the primary probe. On average, eSNPs were significantly associated with 64% of the conditionally correlated probes (Table 3) (multiple testing threshold of 0.05/*n*, where *n* is the number of conditionally correlated probes for a given primary probe). Within the population-based eQTL analysis, these eSNPs were significantly [*P*<4.8e^-4^ (*cis*) and *P*<6.2e^-10^ (*trans*)] associated with their respective conditionally correlated probes in only 3% of cases, implying that in almost all cases they were not identified as contributing to the additive genetic variance of the conditionally correlated probe (Details of conditionally correlated probes are given in Table S2). To further evaluate the impact of the additive effect of the eSNP on the additive genetic variation of the conditionally correlated probes we re-estimated the variance components but included the genotyped eSNP as a fixed covariate in the family based analysis. The mean reduction in 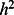 caused by fitting the genotypes of the eSNP is 3.67%. If the conditional correlations were caused by environmental correlations then we would expect no change in the estimate of 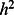. These results demonstrate that a SNP with a *cis*-effect on a particular probe also has *trans*-effects by leading to expression variation of other probes that are within a gene expression network. This approach also allows the identification of links between probes that are caused by genetic effects. This approach also allows the identification of links between probes that are caused by genetic effects.

**Table 3:** Shared additive genetic effects within a pathway of conditionally correlated probes.

Gene	Probe	eSNP	* 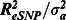	*N* probes with conditional correlations	*N* with significant association with eSNP	Mean change in *h^2^* **
HLA-DRB1	ILMN_1715169	rs9271170	0.99	7	5	3.2 (%)
ERAP2	ILMN_1743145	rs10051637	0.97	5	2	0.7 (%)
MED4	ILMN_1664641	rs943067	0.98	11	7	2.1 (%)
RPS26	ILMN_2209027	rs10876864	0.98	6	4	7.3 (%)
GSTM1	ILMN_1762255	rs11101992	0.98	7	5	2.3 (%)
IRF5	ILMN_2312606	rs6965542	0.99	6	4	3.7 (%)
PAM	ILMN_2313901	rs28092	0.99	6	4	5.5 (%)
ATP13A1	ILMN_2134224	rs2304130	0.97	12	9	4.3 (%)
ZSWIM7	ILMN_3298167	rs1045599	0.98	11	7	4.3 (%)
HBG2	ILMN_2084825	rs766432	0.98	16	10	3.3 (%)

*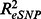 is the proportion of additive variance explained by the eSNP.

** To further demonstrate a genetic causal link between probes, the eSNP from the primary probe was included as a linear covariate in the family based analysis (model [1]). Heritability estimated from this model is conditional on the eSNP genotypes; the difference in 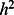 compared to the model not including the eSNP represents the proportion of 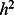 accounted for by the eSNP for the conditionally correlated probes.

### Congruence of pedigree and SNP inference

**Figure 3:**
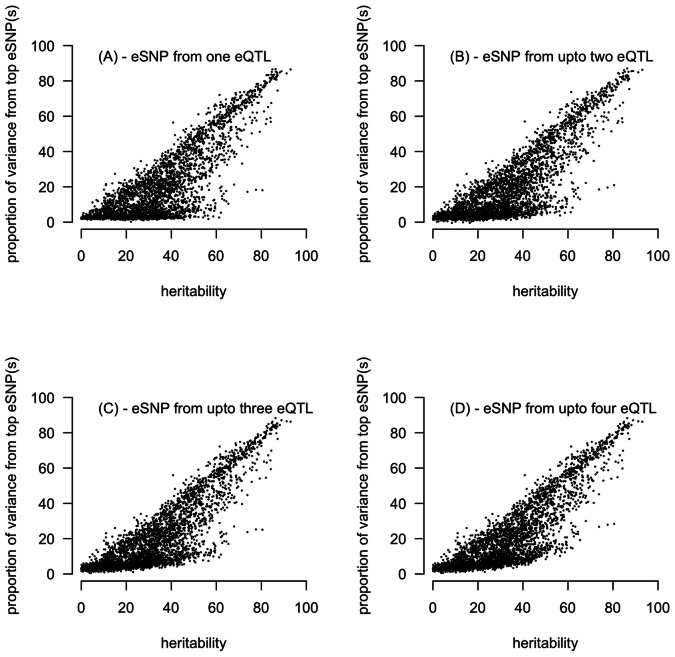
Relationship between narrow-sense heritability estimated from the pedigree against the proportion of variance explained by the top (smallest *P*-) eSNP(s) identified from the additive model eQTL analysis on unrelated individuals. The relationship for the 3,364 probes for which we identified at least one eQTL and a significant heritability estimate is shown. (a) gives the proportion of variance explained by one eQTL and (b) shows the combined proportion of variance explained from up to two eQTL (c) up to three eQTL and (d) up to four eQTL. 3,364 probes had 1+ eQTL, 1,376 had 2+ eQTL, 217 had 3+ eQTL and 76 had 4 eQTL (see Table 2).

There is a strong relationship between narrow-sense heritability estimated from the pedigree and the proportion of variance that is explained by additive eSNPs that can be identified from an eQTL association analysis (Figure 3 and Figure S8). For many transcripts the vast majority of additive variance is accounted for by a few loci, with the proportion of 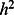 explained by eSNPs greater than 80% for 721 probes (4% of total probes and 21% of probes with an eQTL) (Figure S9). All SNPs accounting for >80% of a probe's 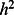 are located within the *cis*-region of the transcription start site (TSS). This is in strong contrast to a mean proportion of variance for *trans*-acting eSNP of 3.2%. Such observations imply that proximal transcription-factor binding sites involved in RNA polymerase II recruitment and subsequent transcription are key components of the regulatory architecture and suggest that distal-acting elements exert a weaker influence.

The two approaches, the first a decomposition of the variance among related individuals and the second an association analysis of SNP genotypes in unrelated individuals, provide independent estimation of the genetic effects influencing transcript levels. We identified additive eQTL for 30% (3,364/11,279) of the probes that had only a significant additive component in the pedigree analysis, which contrasts sharply with only 2% (7/282) of additive eQTL for probes with only significant dominance variance in the pedigree analysis (Table 1). Conversely, 67 dominance eQTL were identified (61 with additive and dominance effects and 6 with over-dominance effects) and 9 replicated for 282 of the probes with just dominance variance in the pedigree analysis, although for the majority of these eQTL a significant additive effect was also identified, reflecting the shared genetic covariance between additive and dominance terms [2].

## Discussion

We have used a complementary analysis of pedigree and SNP data to partition variation for gene expression in whole blood into components of additive, dominance and environmental variation, and have attributed a proportion of additive and dominance variation to specific *cis* and *trans* acting loci. The extent of non-additive genetic variance for gene expression has been investigated in theory [28] and empirically in plant species [14], [29] and model organisms [30]–[32]. This is the first, systematic investigation of non-additive genetic variance influencing RNA transcript variation in humans.

Due to low power to estimate three variance components jointly, we have estimated non-additive and common environmental components in separate models, and show little confounding with estimates of additive variance (Text S1 and Figure S2). Our pedigree design did not allow the separation of dominance variation and that due to epistatic interactions, and our SNP analysis lacked power to detect and replicate specific epistatic interactions. In our pedigree design, epistatic variation is partially confounded with dominance. The inference we draw from the pedigree and SNP analyses are consistent and although we cannot rule out variation due to epistasis, it is unlikely to contribute a large proportion of phenotypic variation. One possibility is that common environmental and non-additive effects are manifesting as the additive component. However, our strong relationship between additive variance estimated from pedigree and SNPs data is not consistent with this hypothesis. It is also possible but unlikely that the variance due to common environmental factors and non-additive genetic factors cancel each other out by chance. Thus the most parsimonious explanation of the results is that additive variance explains most of the observed similarity between relatives and non-additive variance is generally of small magnitude and cannot explain a large proportion of the genetic (and therefore phenotypic) variance. The large-scale additive genetic contribution to phenotypic variance is in line with predictions from theory [4] and is important in the context of understanding the impact of gene expression variance on complex disease. How strong is the relationship between the pedigree and SNP based estimates of additive variation? For heritable probes with a significant eQTL the relationship is very strong, with the mean proportion of the estimate of narrow sense heritability from the pedigree explained by SNPs of 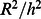 = 0.38 (Figure S9). For many of these probes the identification of SNPs explaining the majority of additive variance, located in *cis*-regions, provides strong support that the underlying molecular mechanisms that influence expression at these transcript positions can be identified through targeted sequencing.

Significant non-additive effects are identified for a few probes; with one particularly interesting finding of the SNP associations showing over-dominance with probe transcript levels. As a component of maintaining genetic variance, over-dominance has been discussed in livestock and model species [33], [34], however, in humans, other than Sickle-Cell Anaemia, few examples of over-dominance have been shown. Whilst the exact mechanism by which rs12313805 acts in an over-dominant manner to influence transcription levels in ETV6 is unknown, non-additive effects at the gene expression level raise the possibility of the contribution of non-additive modes of inheritance to higher order phenotypes such as disease susceptibility. If we examine the total of the components of variation for expression of all probes, then we see that non-genetic factors play the largest role (Figure S3). However, there is strong evidence that for the majority of probes the extent of genetic variation as well as the overlap of genetic effects can differ greatly between tissues [18], [19], [35]. Therefore, it is likely that the components of variation for the same probe measured in other tissues will be different.

Despite the similar inference on mode of gene action from the pedigree and SNP analyses, there appears to be “missing heritability” for many probes (Figure 3 and Figures S8 and S9). Missing heritability for transcript probes is likely to be due to similar factors as that for other complex traits, where there is increasing evidence for large numbers of common SNPs with small effect [7], [36]. For gene expression, one explanation for the occurrence of SNPs with small effects is the impact of an eQTL on the transcription of multiple probes across a pathway or gene network. When we observe probes whose additive variance can almost entirely be attributed to a single locus, what is the impact of that SNP on the transcription of other genes involved in the same pathway? In other words, if 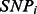 accounts for 90% of 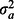 for 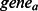, and the product of 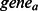 influences transcription at 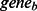, 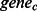 and 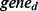, then what effect does 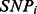 have on 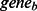, 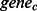 and 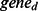
? We have presented an approach that demonstrates a causal genetic relationship between large *cis*-acting eQTL and multiple conditionally correlated probes (Table 3). The majority of such associations would have been missed from a conventional eQTL mapping study due to high significance thresholds imposed when searching for *trans*-associations.

In conclusion, we used a complementary pedigree and SNP-based design and analysis to dissect phenotypic variation for gene expression to inform on the underlying genetic architecture. We show that whilst a small proportion of genetic variance acts in a non-additive manner, the vast majority is additive. We also demonstrate a genetic causal link between eQTL with large *cis*-effects and secondary probes acting within a gene expression network.

## Materials and Methods

### Ethics statement

All participants gave informed consent and the study protocol was approved by the appropriate institutional review boards.

### BSGS dataset

The Brisbane Systems Genetics Study (BSGS) comprises 862 Individuals of European descent from 312 independent families [16]. Families consist of adolescent monozygotic (MZ) and dizygotic (DZ) twins, their siblings, and their parents (Table S3). DNA samples from each individual were genotyped on the Illumina 610-Quad Beadchip by the Scientific Services Division at deCODE Genetics Iceland. After standard QC filters were applied 528,509 SNPs with MAF>1% remained for further analysis. Full details of genotyping procedures are given by Medland *et al.* [37]. Gene expression profiles were generated from whole blood collected with PAXgene TM tubes (QIAGEN, Valencia, CA) using Illumina HT12-v4.0 bead arrays. Expression levels were corrected for batch, sex and age effects using linear models. The Illumina HT-12 v4.0 chip contains 47,323 probes, although some probes are not assigned to RefSeq genes. SNPs within the probe sequence have the potential to lead to spurious associations [38]. We removed any probe which had a genotyped or imputed SNP with MAF>0.05 located within their probe sequence (Illumina manifest file used for probe coordinates). Non-genotyped SNPs were determined by imputing against 1000 Genomes (V1.3; hg19) data. After quality control to remove poorly imputed SNPs we removed a total of 1,027 probes with SNPs in their probe sequence. For the eQTL analysis, any probes where less than 10% of samples had a detection *P*-value>0.05 were removed from the dataset. Of the 24,317 probes retained, the mean call rate of the proportion of samples with detection *P*-values, 0.05 was 97%, implying that relatively little missing data remained within the expression dataset. After removing 6,322 putative and/or not well-characterised genes i.e. probe names starting with HS (n = 1,841), KIAA (n = 158) and LOC (n = 4,323), 17,994 well-characterised probes remained for analysis, which corresponds to 13,486 RefSeq genes. Gene expression quantification and normalisation are described in Powell *et al.* [16]. Analyses of variance components were carried out using the full BSGS dataset of 862 individuals. Mapping of additive and non-additive SNP associations was conducted on a subset BSGS comprising of 339 unrelated individuals. Including the two parents from families with parents and children in BSGS and a randomly chosen individual from families with no parent in BSGS formed the unrelated dataset. We calculated the pairwise Identity-By-State (IBS) from common SNPs (MAF>0.05) to ensure the unrelated dataset contained no genetic relationships greater than would be expected by sampling from an unrelated population (Figure S10) [7], [39], [40].

### CHDWB dataset

The CHDWB study comprises of 139 Caucasian (CHDWB_EA) healthy individuals, between the ages of 26 and 79. Gene expression profiles were generated using RNA extracted from whole blood collected with Tempus tubes (Applied Biosystems, Foster City, CA, USA) and hybridized to Illumina HT12 v3.0 bead arrays. Genotypes were measured using Illumina OmniQuad arrays. Full details of individuals, generation of RNA transcript abundance and genotype calling are given in Qin *et al.* [17]. Gene expression levels were normalized using the same procedures as applied to BSGS data. After quality control filtering there were 312,151 SNPs that overlapped between BSGS and CHDWB datasets (MAF>0.01).

### Estimating variance components from related individuals

We fitted the following mixed linear model:
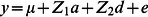
(1)with *y* is an *n×1* vector of gene expression levels. Random additive genetic effects *a* and random dominance effects *d* are related to *y* by incidence matrices *Z_1_* and *Z_2_* respectably. The *n×1* vector *e* contains the error terms. The joint distribution of all variables in [1] is the following:
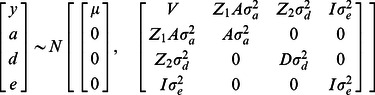
(2)where 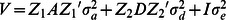. The matrix **A** (*n×n*) is the additive relationship matrix and **D** (*n×n*) is the dominance relationship matrix and ***I*** is an identity matrix. **A** and **D** are calculated from the pedigree relationship information between individuals. Lynch and Walsh [3] detail the calculation of **A** and **D** given pedigree information. For each probe genetic variance components 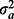, 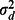 and 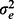, are estimated using an average information REML algorithm [41] implemented in ASREML [42]. Iterations were performed until the minimum variance of the function (−2logL) was less than 1e^-7^. The estimated variance components are expressed as ratios of the total phenotypic variance (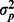) for each model: the additive variance ratio as 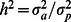, i.e. the heritability, and the dominance genetic variance ratio as 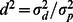. There is not enough information contained between the relative pairs to accurately separate, additional genetic and non-genetic variance components in [1]. In [1] it is assumed that the environmental values of different individuals are independent and uncorrelated with genetic values and so the model does not test for effects such as common family environment. By fitting a model including additive genetic (***a***) and family (***f***) effects to the expression levels of each probe we can estimate the proportions of variance attributable to common environment (variance due to environmental effects shared within a family). Estimates of *h^2^* and *f^2^* (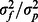) were obtained from
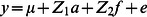
(3)where the joint distribution follows [2], with 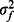 replacing 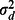 and ***F*** replacing ***D***. Here, ***F*** (*1×n*) is a vector containing family identifiers. For each probe the significance of ***a***, ***d*** and ***f*** estimates were determined by comparing the full model to a reduced model where the relevant term was dropped from the model (Table S4). Full and reduced models were compared using likelihood ratio (LR) tests. From resulting *P*-values a transcript-wise FDR was calculated.

### Estimating SNP effects from unrelated individuals

We tested for association between the 528,509 genotyped SNPs and the normalised expression levels of 17,994 probes using the linear regression functions in PLINK [43]. In order to detect independent eQTL we performed a series of conditional regression analyses. For each probe with an identified eQTL we corrected for the main effects of the top eSNP (SNP with the strongest association) by regressing its genotypes against the expression levels. Residuals from this analysis were then used for second round of eQTL mapping, allowing us to detect independent eQTL. If additional eQTL were identified from this second round of analysis, the process was repeated, correcting for the main effects of the top eSNP from the first and second eQTL using multivariate regression. This process was repeated until either a) no additional significant eQTL were identified or b) four independent eQTL had been identified. *Cis*-eQTLs were defined as associations between SNPs within 1MB of either the 3′ or 5′ end of the TSS. We defined trans-associations as associations involving SNPs elsewhere in the genome. To correct for multiple testing, we controlled the FDR [44] at 0.05: the distribution of observed *P*-values was used to calculate the FDR, by comparing it with the distribution obtained from permuting expression phenotypes relative to genotypes 100 times. At an FDR = 0.05 level, the significance *P*–value thresholds were 4.8e^-4^ (*cis*) and 6.2e^-10^ (*trans*). For probes that were included for series of conditional analyses, the false discovery was again controlled at 0.05 by correcting for the number of running 100 permutations where the top associations were included as conditional main effects. In addition to testing additive effects, we tested for associations that included a dominance component for each SNP by probe. Associations were tested using the –genotypic command in PLINK [43] which fits a 2 degree of freedom joint test for both additive and dominance terms. As described above, we controlled for multiple testing by using an FDR of 0.05 calculated from a 1000 cycle permutation analysis where the permuted phenotype was tested for association using a 2 degrees of freedom model. At an FDR = 0.05 level, the significance *P*–value thresholds for the dominance term (deviation) were 4.1e^-4^ (*cis*) and 4.7e^-10^ (*trans*).

## Supporting Information

Figure S1 Distributions showing the proportion of phenotypic variance attributable to additive genetic (*h^2^*) (a), non-additive genetic (*d^2^*) (b) and common family (*f^2^*) (c) effects. The distributions for all probes (n = 17,994) are shown. Estimates of *h^2^* and *d^2^* were obtained by fitting an ADE model, whist *f^2^* estimates were obtained from a ACE model.
(DOCX)

Figure S2 Relationship between the estimated variance components under full and reduced models.
(DOCX)

Figure S3The cumulative components of phenotypic variance for the 17,994 probes as obtained by fitting an ADE model using family relationship information. Probes are ordered according to the proportion of 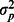 explained by 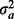.(DOCX)

Figure S4 Distributions of *n* = 17,994 variance components estimated under a null model where the variance component is equal 0 (see Text S2). a) additive variance (Va); b) non-additive variance (Vd); c) common family variance (Vf).
(DOCX)

Figure S5 Distribution of the phenotypic correlations between the parent pairs (n = 71) for the 17,994 probes. The red arrow denotes the mean correlation of the 17 probes showing a significant common environmental effect (Table S1).
(DOCX)

Figure S6 Estimates of the dominance effect (*d*) for 176 SNPs estimated in BSGS and CHDWB_EA samples. SNPS were identified as having a significance dominance effect in BSGS. Red denotes SNPs that replicated at a significance threshold of *p*<2e^-4^ in CHDWB_EA.
(DOCX)

Figure S7 Analysis of the dominance association on chromosome 12 for ILMN_1789596. A 3-marker sliding haplotype window parameterized for just (a) additive or (b) additive and dominance terms. Manhattan plots additive (c) and non-additive (d) association tests using imputed genotype data +/−5MB of rs12313805. Across this region there are no two SNPs with additive effects large enough that should they be in opposite directions, could combine to cause a spurious over-dominance association of the magnitude observe here.
(DOCX)

Figure S8 Relationship between narrow-sense heritability estimated from the pedigree against the proportion of variance explained by the top (smallest *p*-value) eSNP(s) identified from the additive model eQTL analysis on unrelated individuals. This relationship for all probes (n = 17,994) is shown. (a) gives the proportion of variance explained by one eQTL and (b) shows the combined proportion of variance explained from up to two eQTL (c) up to three eQTL and (d) up to four eQTL. 3,364 probes had 1 or more eQTL, 1,376 had 2 or more eQTL, 217 had 3 or more eQTL and 76 had 4 eQTL (see main text table 2).
(DOCX)

Figure S9 Proportion of narrow-sense heritability attributable to the top eSNPs identified from the eQTL analysis. Estimates of *h^2^* are determined from an Additive and non-additive genetic variance model (see methods equation 1), applied to related individuals whilst the proportion of variance explained by eSNPs is estimated from an additive model applied to unrelated individuals. The x-axis shows the proportion of additive variance of each transcript that is explained by eSNP estimated from an independent source.
(DOCX)

Figure S10 Off-diagonal elements from a genomic relationship matrix calculated using 501,279 genome-wide SNPs on 339 individuals.
(DOCX)

Table S1 Information on the 17 probes that have a significant (*p*<1e-4) common family effect. Variance components *h^2^* and *d^2^* were estimated using equation [1] (main text) and *f^2^* using model [3] (main text).
(DOCX)

Table S2Primary and their conditionally correlated probes. 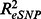 is the variance explained by the eSNP. P-values from the association of the eSNP with conditionally correlated probes are given, with significant associations (multiple testing corrected for using a Bonferroni adjustment) denoted with *. To further demonstrate a genetic causal link between probes, the eSNP from the primary probe was included as a linear covariate in the family based analysis (model [1]). Heritability estimated from this model is conditional on the eSNP genotypes; 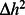 is the difference in 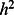 compared to the model not including the eSNP represents the proportion of 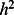 accounted for by the eSNP for the conditionally correlated probes.(DOCX)

Table S3 Summary statistics for the 832 individuals in BSGS. Phenotypic correlations were calculated between pairs of individuals for normalised expression levels in each of the 17,994 probes.
(DOCX)

Table S4 Full and reduced models for variance components.
(DOCX)

Text S1 Confounding between variance component estimates.
(DOCX)

Text S2 Sampling variance of variance components.
(DOCX)
