# Supplementary figures and images for: Congruence of Additive and Non-Additive Effects on Gene Expression Estimated from Pedigree and SNP Data
Source: PLoS Genet. 2013 May 16;9(5):e1003502. doi: 10.1371/journal.pgen.1003502 (PMC3656157; doi:10.1371/journal.pgen.1003502)

a) b)

**
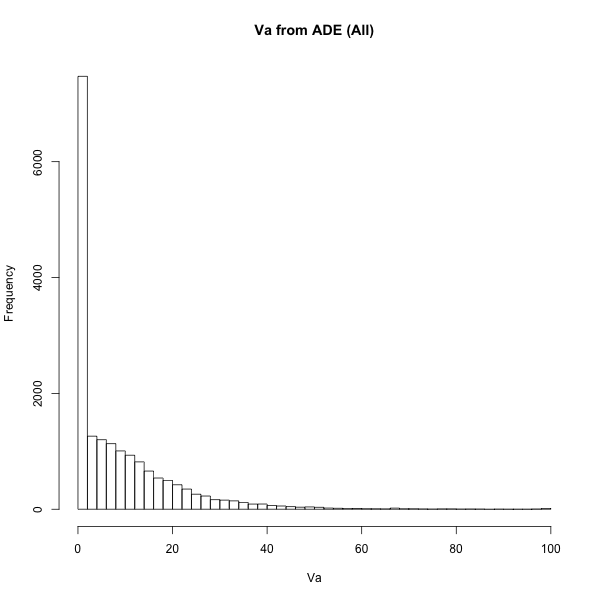
**
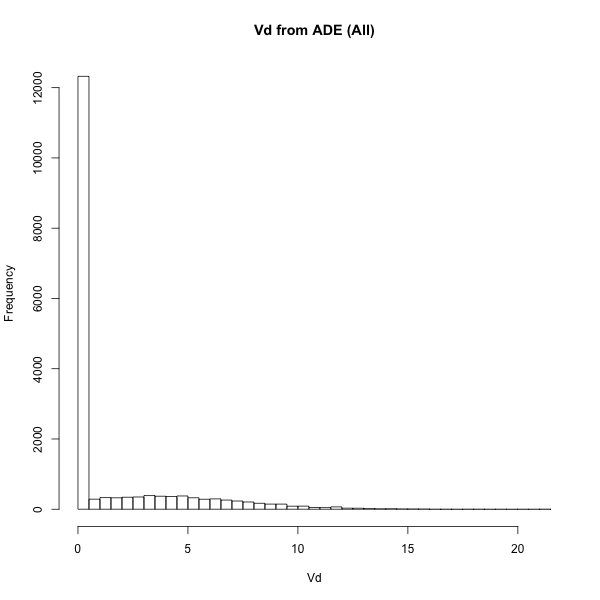


c)


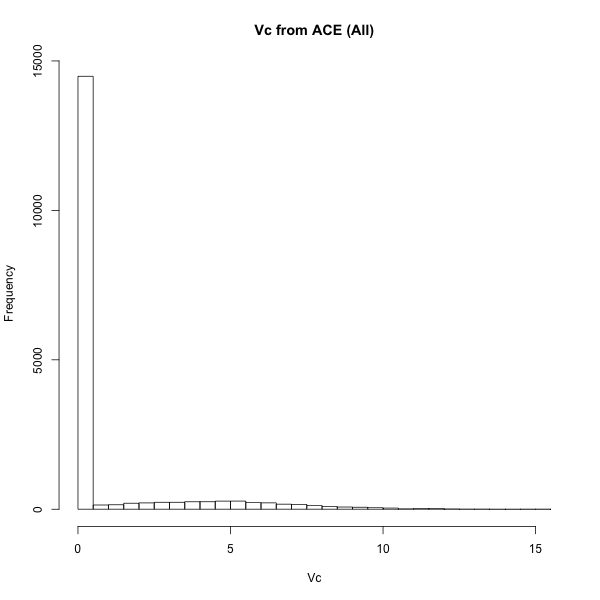

Supplement: Figure S1 — Distributions showing the proportion of phenotypic variance attributable to additive genetic (h2) (a), non-additive genetic (d2) (b) and common family (f2) (c) effects. The distributions for all probes (n = 17,994) are shown. Estimates of h2 and d2 were obtained by fitting an ADE model, whist f2 estimates were obtained from a ACE model. (DOCX) [file pgen.1003502.s001.docx]

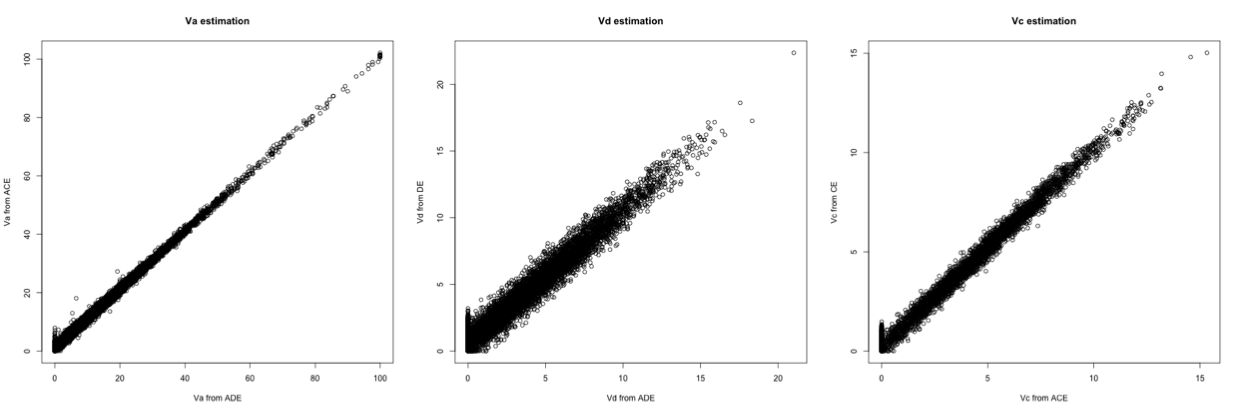

Supplement: Figure S2 — Relationship between the estimated variance components under full and reduced models. (DOCX) [file pgen.1003502.s002.docx]

**
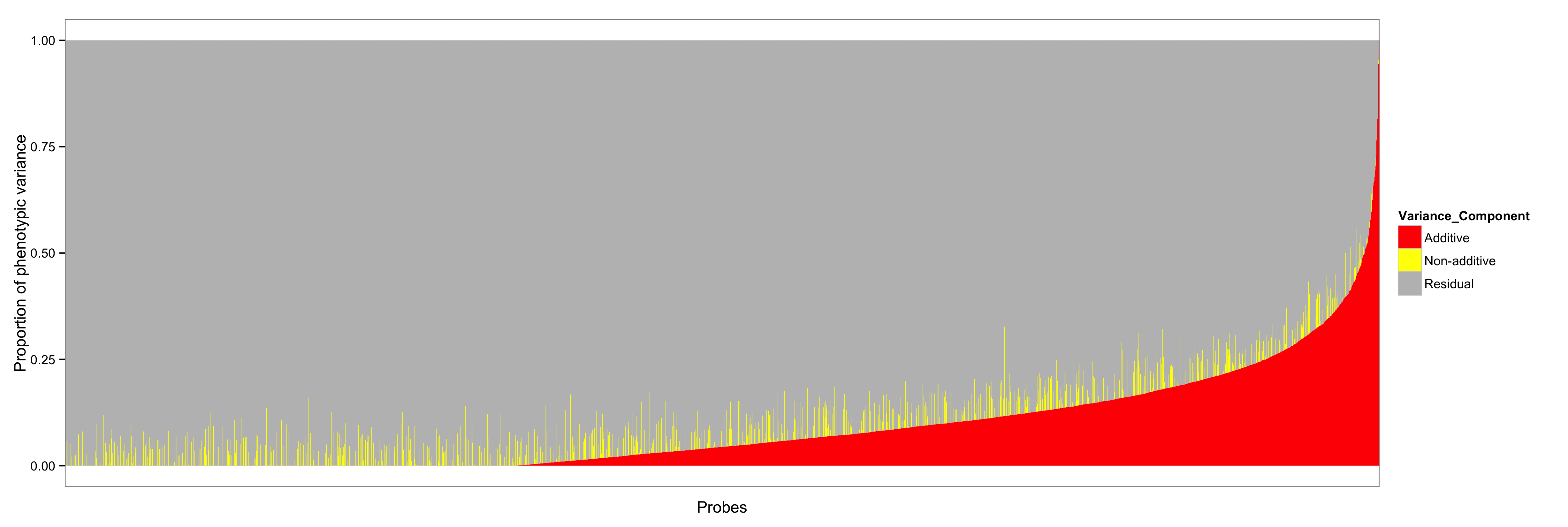
**

Supplement: Figure S3 — The cumulative components of phenotypic variance for the 17,994 probes as obtained by fitting an ADE model using family relationship information. Probes are ordered according to the proportion of explained by . (DOCX) [file pgen.1003502.s003.docx]

a) b)


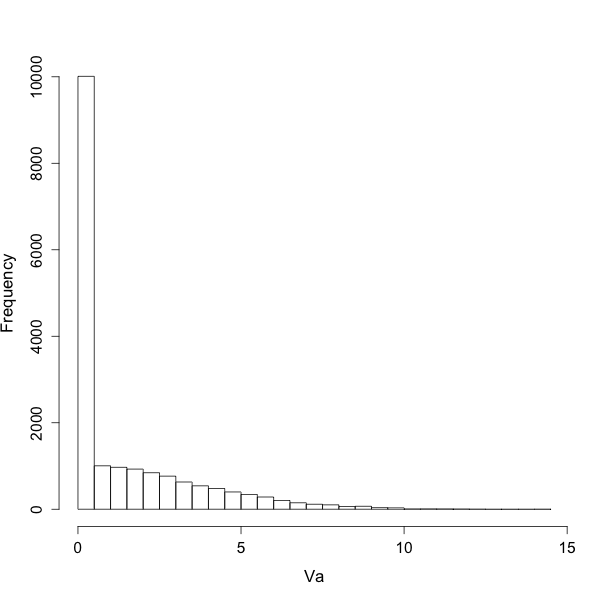

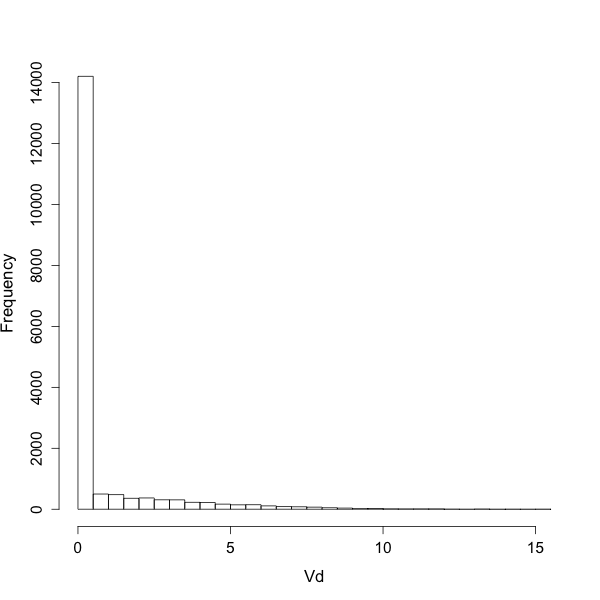


c)


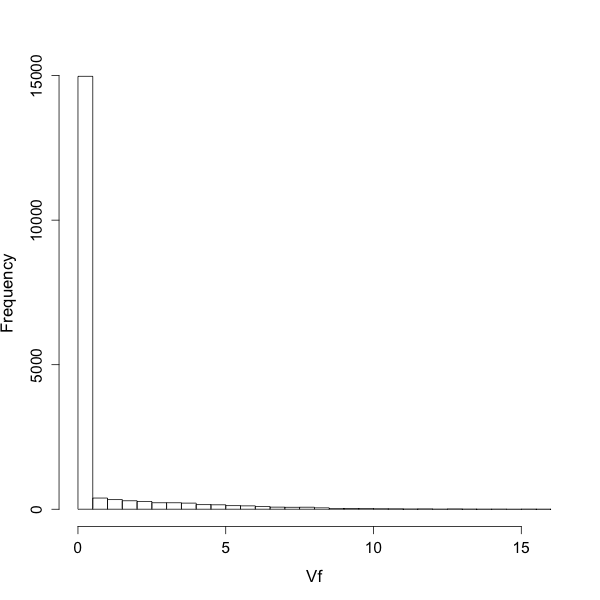

Supplement: Figure S4 — Distributions of n = 17,994 variance components estimated under a null model where the variance component is equal 0 (see Text S2). a) additive variance (Va); b) non-additive variance (Vd); c) common family variance (Vf). (DOCX) [file pgen.1003502.s004.docx]

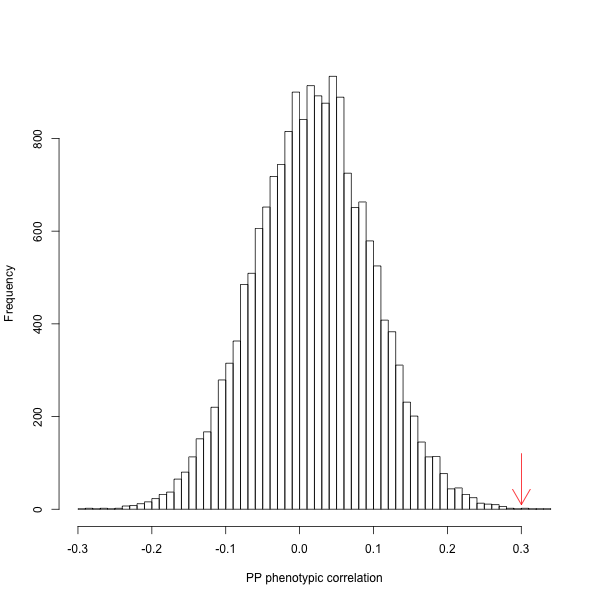

Supplement: Figure S5 — Distribution of the phenotypic correlations between the parent pairs (n = 71) for the 17,994 probes. The red arrow denotes the mean correlation of the 17 probes showing a significant common environmental effect (Table S1). (DOCX) [file pgen.1003502.s005.docx]

**
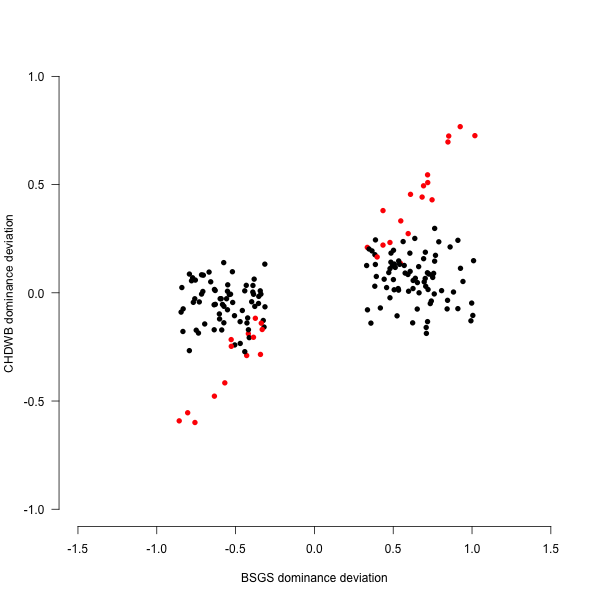
**

Supplement: Figure S6 — Estimates of the dominance effect (d) for 176 SNPs estimated in BSGS and CHDWB_EA samples. SNPS were identified as having a significance dominance effect in BSGS. Red denotes SNPs that replicated at a significance threshold of p<2e-4 in CHDWB_EA. (DOCX) [file pgen.1003502.s006.docx]

a) b)


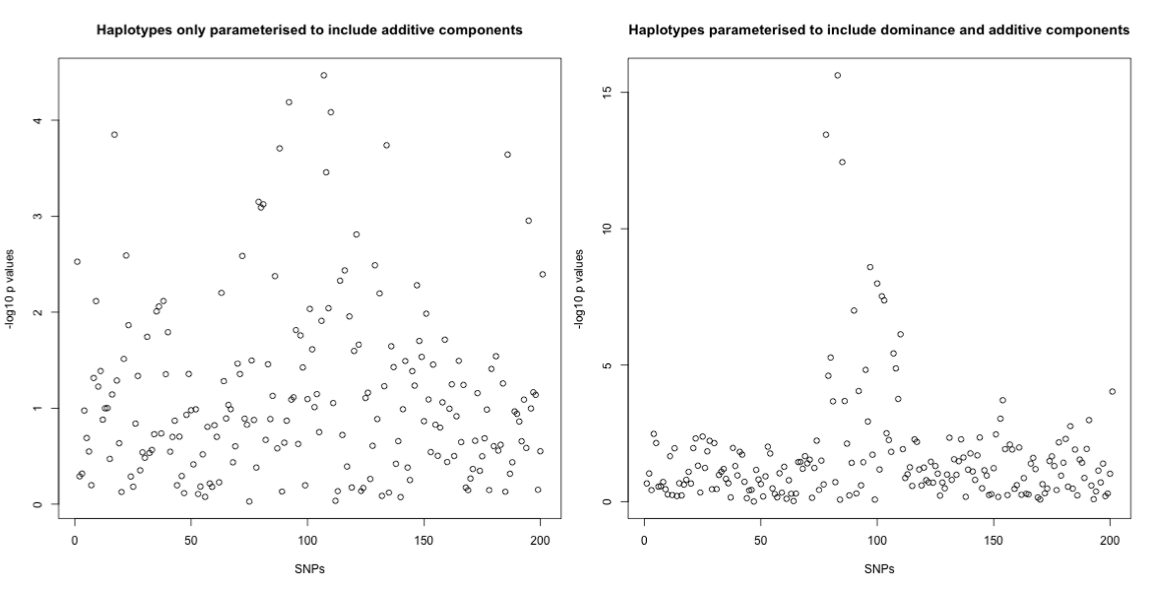


c) d)


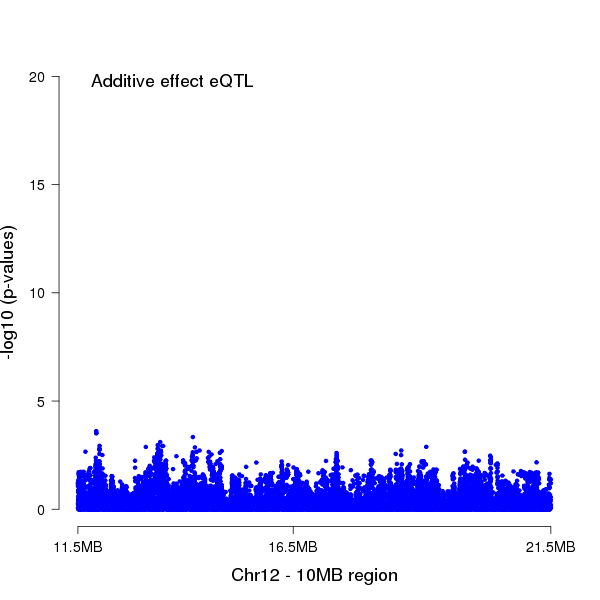

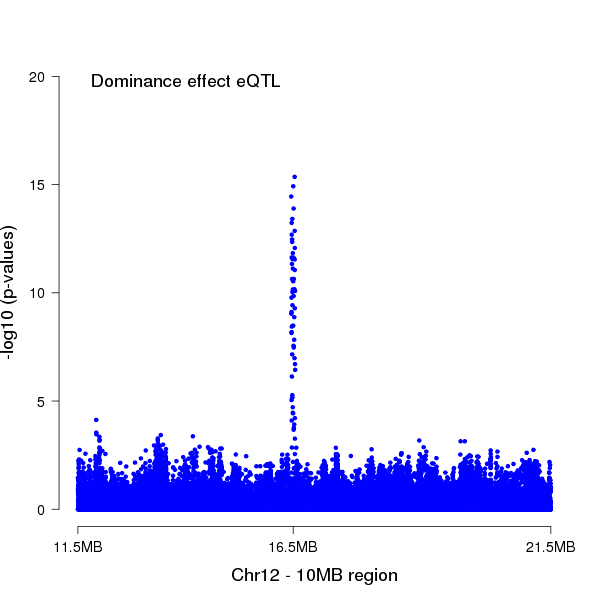

Supplement: Figure S7 — Analysis of the dominance association on chromosome 12 for ILMN_1789596. A 3-marker sliding haplotype window parameterized for just (a) additive or (b) additive and dominance terms. Manhattan plots additive (c) and non-additive (d) association tests using imputed genotype data +/−5MB of rs12313805. Across this region there are no two SNPs with additive effects large enough that should they be in opposite directions, could combine to cause a spurious over-dominance association of the magnitude observe here. (DOCX) [file pgen.1003502.s007.docx]

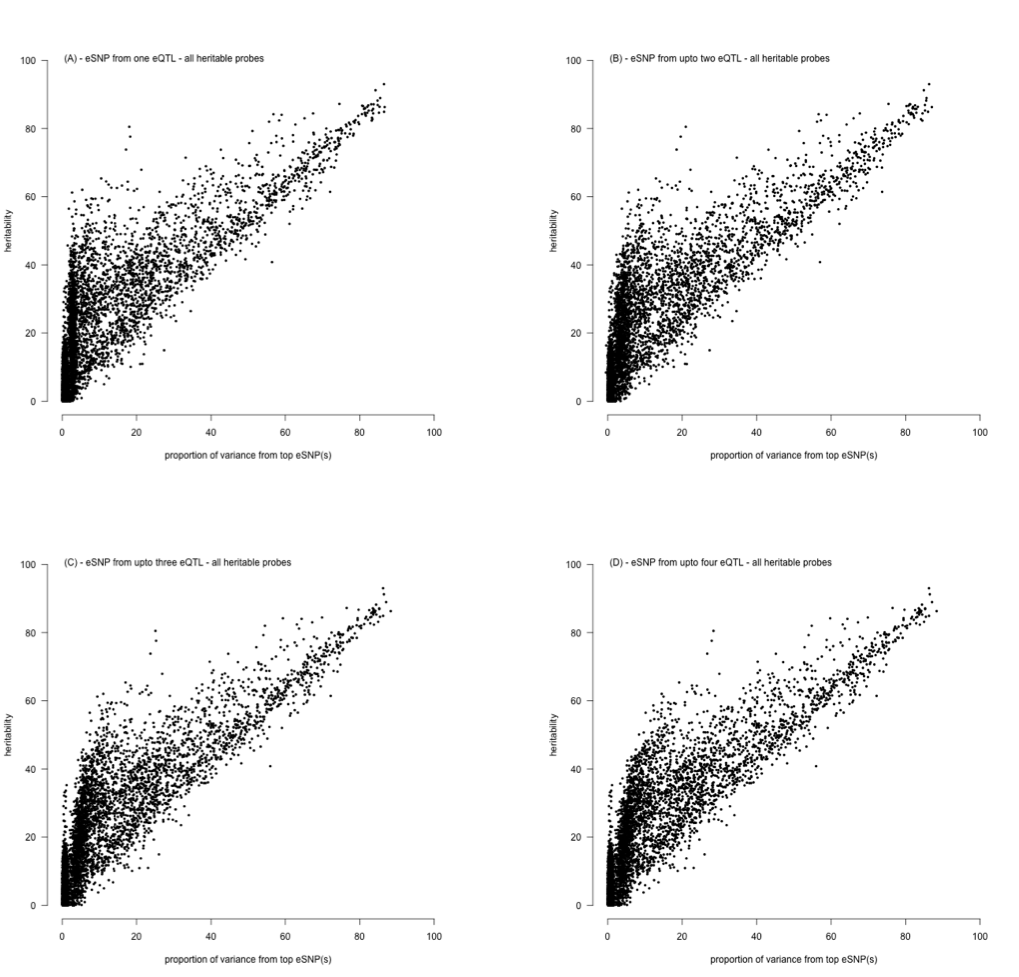

Supplement: Figure S8 — Relationship between narrow-sense heritability estimated from the pedigree against the proportion of variance explained by the top (smallest p-value) eSNP(s) identified from the additive model eQTL analysis on unrelated individuals. This relationship for all probes (n = 17,994) is shown. (a) gives the proportion of variance explained by one eQTL and (b) shows the combined proportion of variance explained from up to two eQTL (c) up to three eQTL and (d) up to four eQTL. 3,364 probes had 1 or more eQTL, 1,376 had 2 or more eQTL, 217 had 3 or more eQTL and 76 had 4 eQTL (see main text table 2). (DOCX) [file pgen.1003502.s008.docx]

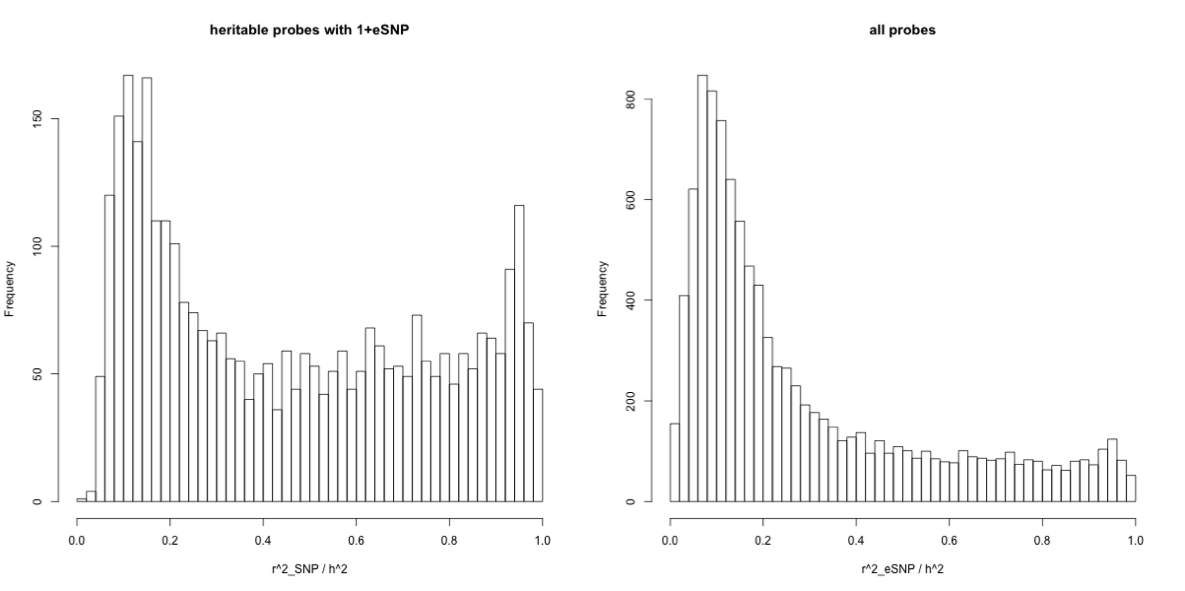

Supplement: Figure S9 — Proportion of narrow-sense heritability attributable to the top eSNPs identified from the eQTL analysis. Estimates of h2 are determined from an Additive and non-additive genetic variance model (see methods equation 1), applied to related individuals whilst the proportion of variance explained by eSNPs is estimated from an additive model applied to unrelated individuals. The x-axis shows the proportion of additive variance of each transcript that is explained by eSNP estimated from an independent source. (DOCX) [file pgen.1003502.s009.docx]

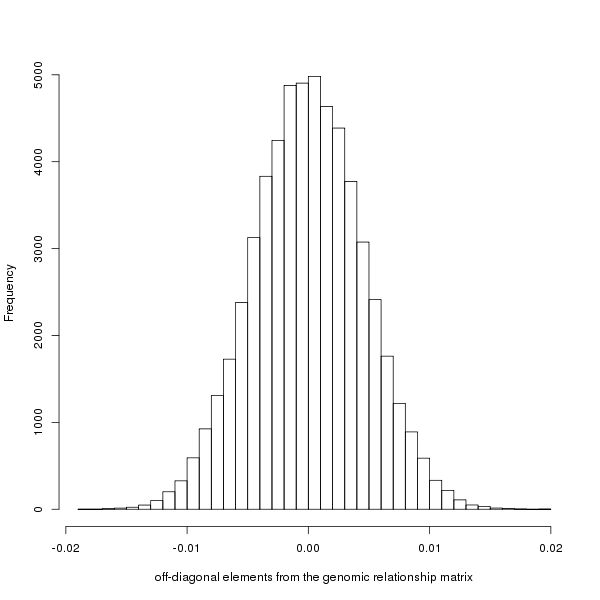

Supplement: Figure S10 — Off-diagonal elements from a genomic relationship matrix calculated using 501,279 genome-wide SNPs on 339 individuals. (DOCX) [file pgen.1003502.s010.docx]
