# Supplementary material for: Congruence of Additive and Non-Additive Effects on Gene Expression Estimated from Pedigree and SNP Data
Source: PLoS Genet. 2013 May 16;9(5):e1003502. doi: 10.1371/journal.pgen.1003502 (PMC3656157; doi:10.1371/journal.pgen.1003502)
Supplement: Table S1 — Information on the 17 probes that have a significant (p<1e-4) common family effect. Variance components h2 and d2 were estimated using equation [1] (main text) and f2 using model [3] (main text). (DOCX) [file pgen.1003502.s011.docx]

**Supplementary Table 1 |** Information on the 17 probes that have a significant (*p* < 1e-4) common family effect. Variance components *h^2^* and *d^2^* were estimated using equation [1] (main text) and *f^2^* using model [3] (main text).

| Gene | Probe | Chr | Position (bb) | *h^2^* | *d^2^* | *f^2^* | Function |
| --- | --- | --- | --- | --- | --- | --- | --- |
| ATRN | ILMN_2355586 | 20 | 3529623 | 4.2 | 1.2 | 16.4 | Proteins involved in the initial immune cell clustering during inflammatory responses that may regulate the chemotactic activity of chemokines. |
| C11ORF63 | ILMN_1709050 | 11 | 122280189 | 3.2 | 0.9 | 15.9 | Uncharacterized protein C11orf63 |
| C16ORF73 | ILMN_1754241 | 16 | 1824080 | 6.8 | 2.3 | 15.7 | Uncharacterized protein C11orf63 |
| CCDC33 | ILMN_1681136 | 15 | 72415460 | 4.7 | 2.7 | 15.6 |  |
| CNTN5 | ILMN_1710289 | 11 | 98932098 | 6.5 | 0.2 | 14.5 | Protein encoded by this gene is a member of the immunoglobulin superfamily, and contactin family, which mediate cell surface interactions during nervous system development |
| DNAL1 | ILMN_1730464 | 14 | 73232375 | 0.0 | 0.0 | 14.2 | Gene encodes an axonemal dynein light chain which functions as a component of the outer dynein arms complex. This complex acts as the molecular motor that provides the force to move cilia in an ATP-dependent manner |
| FAM26E | ILMN_3236080 | 6 | 116839628 | 2.4 | 1.5 | 13.8 |  |
| GKN2 | ILMN_1813688 | 2 | 69172515 | 1.6 | 3.6 | 13.8 |  |
| GRASP | ILMN_1705210 | 12 | 50695586 | 0.7 | 0.7 | 13.7 | Plays a role in intracellular trafficking and contributes to the macromolecular organization of group 1  metabotropic glutamate receptors at synapses |
| ITIH1 | ILMN_1755251 | 3 | 52800872 | 0.0 | 2.6 | 13.6 | The protein encoded by this gene is the heavy chain of a serine protease inhibitor that may serve to carry hyaluronan  in plasma |
| OTC | ILMN_1749114 | 23 | 38156192 | 2.4 | 4.8 | 13.1 | Gene encodes a mitochondrial matrix enzyme |
| RGPD5 | ILMN_2246256 | 2 | 109951178 | 0.0 | 0.9 | 13.0 | Small GTP-binding protein of the RAS superfamily that is associated with the nuclear membrane and is thought to control a variety of cellular functions through its interactions with other proteins |
| SLC6A1 | ILMN_1744191 | 3 | 11055330 | 0.2 | 1.7 | 12.8 | Gene encodes a gamma-aminobutyric acid (GABA) transporter, which removes GABA from the synaptic cleft |
| TCP10L | ILMN_1754656 | 21 | 32870965 | 0.0 | 2.2 | 12.7 | T-complex protein 10 |
| TFAP2A | ILMN_1765574 | 6 | 10506312 | 8.4 | 3.5 | 12.7 | Gene is a transcription factor that binds the consensus sequence 5'-GCCNNNGGC-3'. The encoded protein functions as either a homodimer or as a heterodimer with similar family members. This protein activates the transcription of some genes while inhibiting the transcription of others. |
| TMPRSS13 | ILMN_1671154 | 11 | 117276857 | 2.7 | 0.6 | 12.5 | Gene encodes a member of the type II transmembrane serine protease family |
| TPX2 | ILMN_1796949 | 20 | 29852491 | 6.4 | 2.4 | 12.1 | Spindle assembly factor. Required for normal assembly of mitotic spindles |
