# Supplementary material for: Congruence of Additive and Non-Additive Effects on Gene Expression Estimated from Pedigree and SNP Data
Source: PLoS Genet. 2013 May 16;9(5):e1003502. doi: 10.1371/journal.pgen.1003502 (PMC3656157; doi:10.1371/journal.pgen.1003502)
Supplement: Table S2 — Primary and their conditionally correlated probes. is the variance explained by the eSNP. P-values from the association of the eSNP with conditionally correlated probes are given, with significant associations (multiple testing corrected for using a Bonferroni adjustment) denoted with *. To further demonstrate a genetic causal link between probes, the eSNP from the primary probe was included as a linear covariate in the family based analysis (model [1]). Heritability estimated from this model is conditional on the eSNP genotypes; is the difference in compared to the model not including the eSNP represents the proportion of accounted for by the eSNP for the conditionally correlated probes. (DOCX) [file pgen.1003502.s012.docx]

**Supplementary Table 3** | Primary and their conditionally correlated probes. $R_{eSNP}^{2}$ is the variance explained by the eSNP. P-values from the association of the eSNP with conditionally correlated probes are given, with significant associations (multiple testing corrected for using a Bonferroni adjustment) denoted with *. To further demonstrate a genetic causal link between probes, the eSNP from the primary probe was included as a linear covariate in the family based analysis (model [1]). Heritability estimated from this model is conditional on the eSNP genotypes; $\Delta h^{2}$is the difference in $h^{2}$ compared to the model not including the eSNP represents the proportion of $h^{2}$ accounted for by the eSNP for the conditionally correlated probes.

| **Primary Probes** | | | **Conditionally correlated probes** | | | | | |
| --- | --- | --- | --- | --- | --- | --- | --- | --- |
| Gene | Probe | eSNP | Gene | Probe | Conditional Correlation | *p* value of the eSNP | ${{(R}_{eSNP}^{2}}/{\sigma_{a}^{2})}$  *100 | $\Delta h^{2}$ |
| HLA-DRB1 | ILMN_1715169 | rs9271170 |  |  |  |  |  |  |
|  |  |  | HLA-DRB3 | ILMN_1717261 | 0.64 | 2.78e-13* | 7.29 | -5.91 |
|  |  |  | TMEM154 | ILMN_2088124 | 0.16 | 5.05e-4* | 1.56 | -2.42 |
|  |  |  | NR4A2 | ILMN_1782305 | 0.09 | 1.10e-3* | 1.32 | -4.33 |
|  |  |  | ZNF436 | ILMN_2357781 | 0.37 | 7.72e-4* | 3.44 | -1.65 |
|  |  |  | LST1 | ILMN_1718936 | 0.22 | 9.27e-6* | 4.27 | -4.62 |
|  |  |  | ZNF385C | ILMN_1770400 | 0.07 | 2.95e-2 | 2.68 | 0.00 |
|  |  |  | C12ORF73 | ILMN_3241041 | 0.11 | 1.98e-1 | 1.74 | 0.01 |
| ERAP2 | ILMN_1743145 | rs10051637 |  |  |  |  |  |  |
|  |  |  | ANKRD47 | ILMN_1755588 | 0.33 | 5.27e-4* | 5.91 | -1.91 |
|  |  |  | ERAP1 | ILMN_2336220 | 0.49 | 1.03e-5* | 3.66 | -2.01 |
|  |  |  | MRPS30 | ILMN_1726743 | 0.07 | 1.24e-1 | 0.51 | 0.00 |
|  |  |  | PPM1A | ILMN_1727127 | 0.31 | 1.25e-1 | 0.51 | 0.00 |
|  |  |  | SDR42E1 | ILMN_1798817 | 0.10 | 1.04e-1 | 1.03 | 0.00 |
| MED4 | ILMN_1664641 | rs943067 |  |  |  |  |  |  |
|  |  |  | ABHD14B | ILMN_2227533 | 0.16 | 1.22e-4* | 2.07 | -2.36 |
|  |  |  | BRMS1L | ILMN_1775943 | 0.17 | 9.39e-5* | 4.81 | -3.66 |
|  |  |  | TOP1MT | ILMN_2405628 | 0.28 | 5.87e-4* | 1.69 | -4.45 |
|  |  |  | JAG1 | ILMN_1691376 | 0.34 | 4.97e-4* | 12.8 | -4.18 |
|  |  |  | DOK1 | ILMN_1700086 | 0.09 | 4.03e-4* | 11.7 | -0.28 |
|  |  |  | RAP1GDS1 | ILMN_1687724 | 0.14 | 5.42e-5* | 4.64 | -1.13 |
|  |  |  | PIGM | ILMN_1799860 | 0.18 | 3.62e-4* | 1.74 | -0.91 |
|  |  |  | TIMM23 | ILMN_1679555 | 0.25 | 1.23e-1 | 0.32 | -0.03 |
|  |  |  | RORA | ILMN_2322498 | 0.03 | 1.21e-1 | 0.43 | -0.03 |
|  |  |  | EIF4E3 | ILMN_2225144 | 0.16 | 1.27e-1 | 0.36 | 0.02 |
|  |  |  | FBXO5 | ILMN_1710676 | 0.27 | 1.08e-1 | 0.39 | 0.00 |
| RPS26 | ILMN_2209027 | rs10876864 |  |  |  |  |  |  |
|  |  |  | RPS26L | ILMN_2310703 | 0.81 | 1.24e-98* | 60.8 | -28.6 |
|  |  |  | FBXO11 | ILMN_1678404 | 0.07 | 1.14e-3* | 8.65 | -8.21 |
|  |  |  | C3ORF59 | ILMN_1700967 | 0.16 | 1.8e-3* | 1.95 | -4.02 |
|  |  |  | MSL2 | ILMN_1766859 | 0.08 | 1.3e-3* | 1.72 | -2.90 |
|  |  |  | SDCCAG3 | ILMN_1803997 | 0.06 | 1.84e-1 | 0.21 | 0.00 |
|  |  |  | NUP88 | ILMN_1734826 | 0.03 | 3.26e-1 | 0.11 | -0.04 |
| GSTM1 | ILMN_1762255 | rs11101992 |  |  |  |  |  |  |
|  |  |  | GSTM2 | ILMN_2201580 | 0.65 | 1.17e-14* | 8.26 | -7.45 |
|  |  |  | C18ORF2 | ILMN_3250066 | 0.34 | 2.16e-3* | 9.61 | -1.31 |
|  |  |  | MESP2 | ILMN_1751911 | 0.04 | 6.40e-4* | 9.38 | -2.36 |
|  |  |  | FLJ23152 | ILMN_1696243 | 0.26 | 3.25e-3* | 10.2 | -3.43 |
|  |  |  | OPRM1 | ILMN_1803261 | 0.16 | 3.50e-3* | 5.34 | -1.92 |
|  |  |  | FCRL4 | ILMN_1738517 | 0.05 | 1.24e-1 | 2.03 | -0.02 |
|  |  |  | ARHGAP24 | ILMN_1775441 | 0.02 | 1.27e-1 | 0.98 | 0.00 |
| IRF5 | ILMN_2312606 | rs6965542 |  |  |  |  |  |  |
|  |  |  | TNPO3 | ILMN_1683811 | 0.43 | 1.55e-10* | 5.78 | -9.96 |
|  |  |  | PHF20 | ILMN_1813657 | 0.17 | 1.24e-3* | 1.29 | -2.91 |
|  |  |  | FABP5L3 | ILMN_2217574 | 0.32 | 2.51e-3* | 11.7 | -3.64 |
|  |  |  | QTRT1 | ILMN_1780153 | 0.25 | 3.95e-3* | 1.02 | -0.72 |
|  |  |  | RAB20 | ILMN_1708881 | 0.19 | 1.08e-1 | 0.74 | -0.18 |
|  |  |  | IFITM4P | ILMN_1770071 | 0.04 | 1.01e-1 | 1.41 | 0.00 |
| PAM | ILMN_2313901 | rs28092 |  |  |  |  |  |  |
|  |  |  | RAB23 | ILMN_2346997 | 0.08 | 4.98e-4* | 3.48 | -7.95 |
|  |  |  | SLCO4C1 | ILMN_1686464 | 0.18 | 1.44e-3* | 1.53 | -4.86 |
|  |  |  | OR10H3 | ILMN_1731314 | 0.29 | 1.22e-3* | 8.73 | -11.3 |
|  |  |  | HELT | ILMN_1693843 | 0.10 | 2.33e-3* | 9.45 | -8.14 |
|  |  |  | MAPK8IP3 | ILMN_1811574 | 0.13 | 1.16e-1 | 0.52 | -0.17 |
|  |  |  | NCOA1 | ILMN_2335198 | 0.26 | 1.26e-1 | 0.36 | -0.34 |
| ATP13A1 | ILMN_2134224 | rs2304130 |  |  |  |  |  |  |
|  |  |  | C11ORF35 | ILMN_1652602 | 0.07 | 7.99e-4* | 4.95 | -6.82 |
|  |  |  | CLCN5 | ILMN_1690327 | 0.20 | 1.88e-3* | 3.69 | -7.34 |
|  |  |  | CRLF2 | ILMN_1767573 | 0.24 | 1.44e-3* | 1.72 | -2.86 |
|  |  |  | LAD1 | ILMN_1728215 | 0.08 | 2.81e-4* | 6.92 | -7.31 |
|  |  |  | NCSTN | ILMN_1735180 | 0.02 | 2.43e-4* | 1.09 | -1.98 |
|  |  |  | HRB | ILMN_2196734 | 0.34 | 5.39e-4* | 5.39 | -4.87 |
|  |  |  | MLXIP | ILMN_1693987 | 0.21 | 4.41e-4* | 9.04 | -12.3 |
|  |  |  | RC1P8 | ILMN_2342068 | 0.14 | 4.12e-3* | 5.10 | -5.12 |
|  |  |  | NCSTN | ILMN_1735180 | 0.10 | 2.43e-3* | 1.07 | -1.01 |
|  |  |  | SLC25A19 | ILMN_1666553 | 0.16 | 1.04e-1 | 0.28 | -0.05 |
|  |  |  | IRF1 | ILMN_1708375 | 0.06 | 2.41e-2 | 0.63 | 0.00 |
|  |  |  | KRT8P9 | ILMN_3191922 | 0.03 | 2.26e-2 | 4.41 | -2.14 |
| ZSWIM7 | ILMN_3298167 | rs1045599 |  |  |  |  |  |  |
|  |  |  | SARM1 | ILMN_1746265 | 0.38 | 7.08e-5* | 2.07 | -6.76 |
|  |  |  | CECR4 | ILMN_3177532 | 0.25 | 4.53e-4* | 10.6 | -16.7 |
|  |  |  | HSPD1 | ILMN_1784367 | 0.14 | 2.39e-6* | 2.88 | -4.51 |
|  |  |  | MTR | ILMN_1670801 | 0.03 | 3.52e-4* | 1.56 | -3.46 |
|  |  |  | C22ORF32 | ILMN_1706859 | 0.16 | 5.22e-5* | 2.31 | -4.82 |
|  |  |  | AMY2B | ILMN_2073157 | 0.18 | 3.38e-4* | 1.64 | -2.47 |
|  |  |  | RALGPS1 | ILMN_1674135 | 0.06 | 5.03e-4* | 1.55 | -3.61 |
|  |  |  | ZSWIM3 | ILMN_2283196 | 0.14 | 1.02e-1 | 0.33 | -1.98 |
|  |  |  | MIF | ILMN_1807074 | 0.12 | 1.98e-2 | 0.71 | -0.18 |
|  |  |  | NELL2 | ILMN_1725417 | 0.05 | 1.12e-1 | 0.34 | -2.45 |
|  |  |  | PPP2R1A | ILMN_1810467 | 0.31 | 1.05e-1 | 0.33 | -0.36 |
| HBG2 | ILMN_2084825 | rs766432 |  |  |  |  |  |  |
|  |  |  | SIAH1 | ILMN_2380566 | 0.13 | 4.7e-10* | 4.78 | -6.81 |
|  |  |  | PKDREJ | ILMN_1673234 | 0.19 | 2.24e-3* | 9.06 | -12.8 |
|  |  |  | DYNC1H1 | ILMN_1780302 | 0.20 | 3.70e-4* | 1.49 | -2.36 |
|  |  |  | ARCN1 | ILMN_1699703 | 0.14 | 9.51e-4* | 1.32 | -3.64 |
|  |  |  | RIPK1 | ILMN_2119535 | 0.31 | 8.16e-5* | 1.91 | -2.47 |
|  |  |  | ZNFX1 | ILMN_1745148 | 0.04 | 4.48e-4* | 1.48 | -3.67 |
|  |  |  | WDR68 | ILMN_1706706 | 0.11 | 6.29e-4* | 1.48 | -2.98 |
|  |  |  | EHD1 | ILMN_1651832 | 0.16 | 3.43e-4* | 1.53 | -2.71 |
|  |  |  | PTPN6 | ILMN_1738675 | 0.22 | 3.66e-4* | 1.51 | -3.78 |
|  |  |  | NCOR2 | ILMN_2340052 | 0.19 | 2.13e-4* | 1.42 | -4.94 |
|  |  |  | CA1 | ILMN_1652431 | 0.25 | 7.73e-2 | 0.23 | -0.27 |
|  |  |  | ACTN4 | ILMN_1725534 | 0.03 | 2.39e-2 | 0.61 | -1.01 |
|  |  |  | FAM178A | ILMN_3233135 | 0.03 | 1.06e-1 | 0.33 | -1.27 |
|  |  |  | KDELR1 | ILMN_2130411 | 0.15 | 1.04e-1 | 0.34 | 0.00 |
|  |  |  | HIST1H3B | ILMN_2222163 | 0.17 | 1.04e-1 | 4.23 | -3.87 |
|  |  |  | MRPL38 | ILMN_1719656 | 0.01 | 1.14e-1 | 0.54 | -0.41 |
