# Supplementary material for: Congruence of Additive and Non-Additive Effects on Gene Expression Estimated from Pedigree and SNP Data
Source: PLoS Genet. 2013 May 16;9(5):e1003502. doi: 10.1371/journal.pgen.1003502 (PMC3656157; doi:10.1371/journal.pgen.1003502)
Supplement: Table S3 — Summary statistics for the 832 individuals in BSGS. Phenotypic correlations were calculated between pairs of individuals for normalised expression levels in each of the 17,994 probes. (DOCX) [file pgen.1003502.s013.docx]

**Supplementary Table 2 |** Summary statistics for the 832 individuals in BSGS. Phenotypic correlations were calculated between pairs of individuals for normalised expression levels in each of the 17,994 probes.

|  | | | **Shared coefficients** | | | **Mean phenotypic correlation for 17,994 probes (sd)** |
| --- | --- | --- | --- | --- | --- | --- |
| **Relationship pair** | **Code** | **N pairs** | **A** | **D** | **F** |  |
| **Monozygotic twins** | MZ | 78 | 1 | 1 | 1 | 0.182 (0.089) |
| **Dizygotic twins** | DZ | 206 | 0.5 | 0.25 | 1 | 0.097 (0.082) |
| **Siblings** | SIB | 343 | 0.5 | 0.25 | 1 | 0.089 (0.084) |
| **Parent – Offspring** | PO | 425 | 0.5 | 0 | 1 | 0.092 (0.081) |
| **Parent – Parent** | PP | 71 | 0 | 0 | 1 | 0.017 (0.075) |
