# Supplementary material for: Congruence of Additive and Non-Additive Effects on Gene Expression Estimated from Pedigree and SNP Data
Source: PLoS Genet. 2013 May 16;9(5):e1003502. doi: 10.1371/journal.pgen.1003502 (PMC3656157; doi:10.1371/journal.pgen.1003502)
Supplement: Table S4 — Full and reduced models for variance components. (DOCX) [file pgen.1003502.s014.docx]

**Supplementary Table 4 |** Full and reduced models for variance components

| Variance component | Full model | Reduced model |
| --- | --- | --- |
| A | $y={\mu+Z}_{1}a+Z_{2}d+e$ | $y=\mu+Z_{2}d+e$ |
| D | $y={\mu+Z}_{1}a+Z_{2}d+e$ | $y={\mu+Z}_{1}a+e$ |
| F | $y={\mu+Z}_{1}a+Z_{2}f+e$ | $y={\mu+Z}_{1}a+e$ |
