# Supplementary material for: Congruence of Additive and Non-Additive Effects on Gene Expression Estimated from Pedigree and SNP Data
Source: PLoS Genet. 2013 May 16;9(5):e1003502. doi: 10.1371/journal.pgen.1003502 (PMC3656157; doi:10.1371/journal.pgen.1003502)
Supplement: Text S1 — Confounding between variance component estimates. (DOCX) [file pgen.1003502.s015.docx]

**Supplementary Note 1**

**Confounding between variance component estimates**

One potential problem when estimating variance component $\sigma_{a}^{2}$, $\sigma_{d}^{2}$ and $\sigma_{f}^{2}$ is that estimates may be confounded due to correlation in the off-diagonals of the relationship matrices **A**, **D**, and **F**. We attempted to evaluate the potential levels of confounding by running a series of full and reduced models and comparing the estimates of the variance components (supplementary figure 2). The following series of models were run;

1. Comparison of $\sigma_{a}^{2}$ in the presence and absence of a non-additive component.

Full: $y={\mu+Z}_{1}a+Z_{2}d+e$

Reduced: $y={\mu+Z}_{1}a+e$

2. Comparison of $\sigma_{d}^{2}$ in the presence and absence of an additive component.

Full: $y={\mu+Z}_{1}a+Z_{2}d+e$

Reduced $y=\mu+Z_{2}d+e$

3. Comparison of $\sigma_{f}^{2}$ in the presence and absence of an additive component.

Full: $y={\mu+Z}_{1}a+Z_{2}f+e$

Reduced: $y={\mu+Z}_{1}f+e$
