# Supplementary material for: Congruence of Additive and Non-Additive Effects on Gene Expression Estimated from Pedigree and SNP Data
Source: PLoS Genet. 2013 May 16;9(5):e1003502. doi: 10.1371/journal.pgen.1003502 (PMC3656157; doi:10.1371/journal.pgen.1003502)
Supplement: Text S2 — Sampling variance of variance components. (DOCX) [file pgen.1003502.s016.docx]

**Supplementary Note 2**

**Sampling variance of variance components**

Here we have estimated variance components for 17,994 transcripts using models [1] and [3] (online methods). The sampling variance of these variance estimates provides information on the expected distributions of estimates under assumptions of known population parameters. Knowledge of the expected sampling variance of each parameter is useful as it allows us to compare the observed distribution of estimates against the expected distribution under assumptions of the true population values being equal to 0. Calculating the expected sampling variances of genetic ($\sigma_{a}^{2}$ and $\sigma_{d}^{2}$) and non-genetic ($\sigma_{f}^{2}$) variances estimated from related individuals is relatively straightforward when estimates are made from simple relationship pairings (i.e. MZ-DZ, Sibling pairs, Parent-Offspring)[^1^](#_ENREF_1)^,^[^2^](#_ENREF_2). However, in more complex pedigrees, such as BSGS, algebraic methods are not available to calculate the expected sampling variance. To explore the expected sampling variance of the parameter estimates in our data we conducted a series of simulation studies to calculate the expected distribution of estimates under the assumption that the true value of the variance component equals 0, whilst the remaining model parameters equal their empirical mean. For example, to estimate the sampling variance of $\sigma_{a}^{2}$, which is estimated using equation [1] (online methods), matrices from the right side of equation [2] were sampled from an appropriate Wishart distribution (see below), with $y$ a sampled from a random normal distribution, and $d$ and $e$ sampled from the empirical estimates for the 17,994 probes. Variance components for the simulated data were obtained by REML[^3^](#_ENREF_3). This process was repeated 100,000 times and the sampling variance calculated from the estimates of $\sigma_{a}^{2}$. A similar approach was used to calculate the sampling variance of $\sigma_{d}^{2}$ and $\sigma_{f}^{2}$, but with *a* sampled from the empirical values and model [1] and [3] used respectively.

**Simulating matrices using the Wishart distribution**

Here we simulated matrices from a Wishart distribution resulting in an *n x n* matrix, where *n* is the number of individuals, and the off diagonals have a built in correlation structure pertaining to the relationship matrix[^4^](#_ENREF_4), hereon denoted **U**, that is being simulated (either **A**, **D** or **F**). The resulting matrix is positive definite.

*Observed relationship matrix*

To generate a matrix having a Wishart distribution, we start with the relationship matrix **U** equal a matrix with *n* rows and *n* columns. From **U** we calculate **W** as, **W=U’U**. Next we perform a Cholesky decomposition of **W**, so that **W=TT’**, where **T** is the lower triangular matrix.

*Wishart distribution*

First an *n* by *n* matrix, **M** is formed with elements populated by random normal deviates generated by the *rnorm* function in R $\left( \mu=0, \sigma^{2}=1 \right)$. A vector **I**, of length *n* is generated with elements drawn from a Chi-squared distribution (*rchisq* function in R) with *n – i* degrees of freedom where *i* is the element in the vector; i.e. element 1 has *n – 1* degrees of freedom, element 2 has *n – 2* degrees of freedom and so on. Element *n* has 1 degree of freedom.

Next an *n* by *n* matrix **Z** is formed. When *i* = 1 the diagonal element is calculated by;

$$Z_{i,i}=I_{i}*I_{i}$$

when *i* > 1 diagonal elements are calculated by;

$$Z_{i,i}=I_{i}*I_{i}+\sum_{j=1}^{i-1} M_{i,j}*M_{i,j}$$

When *i* = 1 off diagonal elements of **Z** are calculated by;

$$Z_{i,j}=Z_{j,i}=M_{i,j}*I_{i}$$

and when *i* > 1 off diagonal elements are calculated by;

$$Z_{i,j}=Z_{j,i}=M_{i,j}*I_{i}+\sum_{j=1}^{i-1} M_{j,i}*M_{i,j}$$

A final matrix, **X**, is the simulated random Wishart matrix based on the relationships contained within **U**, and is calculated by;

**X = TZT’**

**References**

1. Visscher, P.M., Benyamin, B. & White, I. The use of linear mixed models to estimate variance components from data on twin pairs by maximum likelihood. *Twin Res* **7**, 670-4 (2004).

2. Lynch, M. & Walsh, B. *Genetics and Analysis of Quantitative Traits*, (Sinauer Assoc, 1997).

3. Gilmour, A.R., Gogel, B.J., Cullis, B.R., Welham, S.J. & Thompson, J.R. *ASReml User Guide Release 3.0*, (VSN International Ltd, Hemel Hempstead, 2009).

4. Meyer, K. Bias and sampling covariances of estimates of variance components due to maternal effects. *Genetics Selection Evolution* **24**, 487-509 (1992).
